# Cefepime-Induced Encephalopathy in a High-Risk Patient With Renal Insufficiency and Cirrhosis

**DOI:** 10.7759/cureus.18767

**Published:** 2021-10-14

**Authors:** Andrew J Ortega, S. Reshad Ghafouri, Lynn Vu, Brian Edwards, Nils Nickel

**Affiliations:** 1 Internal Medicine, Texas Tech University Health Sciences Center El Paso Paul L. Foster School of Medicine, El Paso, USA; 2 Internal Medicine, Texas Tech University Health Sciences Center El Paso, El Paso, USA

**Keywords:** altered mental status evaluation, cefepime induced neurotoxicity, non-convulsive status epilepticus, live cirrhosis, chronic kidney disease (ckd)

## Abstract

Cefepime is a fourth-generation, cephalosporin antibiotic commonly used as a first-line empirical treatment in a wide range of bacterial infections. It is predominantly excreted renally; therefore, a reduction in kidney function allows for the accumulation of cefepime to potentially toxic levels. Here we present a case of cefepime-induced encephalopathy (CIE) in a 67 years old male patient with advanced-stage renal insufficiency and cirrhosis who was admitted to our hospital for altered mental status (AMS). The patient was initially treated for hepatic encephalopathy (HE) given an elevated ammonia level (105 µg/dL), which had significantly improved. He was also placed on intravenous (IV) cefepime for *Pseudomonas* bacteremia. Four days later, the patient became drowsy and confused. A detailed workup for secondary causes of AMS was performed however no significant acute abnormalities were detected. The ammonia level remained within the normal range. There was no acute intracranial pathology reported on a head computerized tomography (CT). Furthermore, an electroencephalograph (EEG) was obtained which showed generalized periodic discharge with a tri-phasic wave pattern suggesting non-convulsive status epilepticus (NCSE). CIE was suspected at that point and cefepime administration was stopped. Following cefepime discontinuation, there was a remarkable improvement in the patient’s mental status for several days after cefepime discontinuation that supported the diagnosis of CIE in our patient.

Although the exact pathophysiology is unclear, CIE should be suspected in elderly patients, patients with renal dysfunction, and critical illness. Meanwhile, liver dysfunction can be an additional risk factor for CIE as it increases the permeability of the blood-brain barrier (BBB), altered neurotransmission, and neuro-inflammation.

## Introduction

Cefepime is a fourth-generation, parenteral cephalosporin antibiotic, commonly used as a first-line empirical treatment for severe pneumonia or septicemia [1]. There has been an increase in the use of cefepime due to the popularity of its predecessors (the third-generation cephalosporin), the emergence of multidrug-resistant bacteria, and the proven clinical efficacy in various studies [2,3]. Cefepime is predominantly excreted renally (85%), thus a reduction in kidney function causes a proportional reduction of cefepime clearance that allows the accumulation of cefepime in the body to a potentially toxic level. Hence, treatment with cefepime in patients with advanced renal failure that is not really dosed can lead to the development of CIE manifesting mainly as confusion, seizures, myoclonus, or coma. Therefore, in clinical practice, a strong recommendation is made to adjust cefepime dose renally to prevent such toxicity [4].

Additional risk factors for CIE include pre-existing neurological disease, critical illness, altered blood-brain barrier (BBB), and elderly age. The CIE can also occur in patients with normal renal function but the incidence is still low [5,6]. According to Food and Drug Administration (FDA), cefepime update of ongoing safety review, <1% of cefepime is metabolized in the liver; however, a dose adjustment in patients with liver dysfunction is usually not recommended [7].

In this report, we present a case of a patient with stage 4 chronic kidney disease (CKD) along with advanced liver disease (cirrhosis), who received cefepime resulted in altered mental status (AMS) with presumed CIE. The aim of this report is to demonstrate the difficulty of diagnosing CIE in a patient with multiple comorbidities, particularly renal insufficiency, and hepatic dysfunction. Meanwhile, we performed a comprehensive literature review to highlight some important epidemiological findings of CIE such as estimated incidence, clinical features, and treatment options.

## Case presentation

A 67-year-old male patient with a well-known history of congestive heart failure (CHF), decompensated cirrhosis, stage IV CKD, and morbid obesity (BMI 63.4) was brought to our institution due to new-onset AMS. At baseline, the patient was fully alert and oriented with a GCS of 15 per family report. He was febrile (38.4^o^C), tachycardic (HR > 100 bpm), and hypoxic requiring 4L of supplemental oxygen upon arrival.

On exam, the patient was alert but confused unable to follow commands. He had bilateral scleral icterus. The cardiopulmonary examination was remarkable for decreased breath sounds in the left lower lobe, with an irregularly irregular heart rhythm consistent with new-onset atrial fibrillation (AF). He was significantly volume overloaded, with a 2 plus lower extremity pitting edema. The patient had acute hypercapnic respiratory acidosis on an arterial blood gas (ABG). He had significant leukocytosis (WBC 14.31 x 10^3^/µL ) with macrocytic anemia (Hgb 9.5g/dL, MCV 107 fL) on CBC. His INR was also elevated to 7.2 (0.8-1.2). A CMP showed hyperkalemia (6.2 mEq/L), with an increased creatinine level (BUN 51 mg/dL, Cr 2.8 mg/dL) which was consistent with acute renal injury in the setting of CKD. Other notable serum chemistry values were elevated pro B-type natriuretic peptide (proBNP) of 16,500 pg/mL, hyperbilirubinemia (4.8 mg/dL), lactic acidosis (3.2 mEq/L), and hyperammonemia (105 µg/dL). Baseline electrocardiography (ECG) was obtained, which showed AF with a rapid ventricular response (116 beats per minute). A chest x-ray revealed complete opacification of the left hemi-thorax due to a combination of both pleural effusion and atelectasis. There was no acute intracranial pathology reported on head CT. The patient’s AMS was thought to be multifactorial at the time of admission including hepatic encephalopathy (HE), sepsis, and acute hypercapnic respiratory failure.

 The patient was started on non-invasive positive pressure ventilation with supplemental oxygen, lactulose, IV vancomycin with piperacillin-tazobactam (zosyn). Within 48 hours of admission, the patient’s respiratory and mental status showed significant improvement. Due to Pseudomonas aeruginosa bacteremia and worsening renal function, zosyn was changed to IV cefepime. Four days after cefepime initiation, the patient was noted to have decreased in mentation; he became more confused with agitation episodes that worsened over two days and began falling into a state of stupor unable to communicate and follow commands. At that time, the differential diagnoses for the altered mentation narrowed to seizures, HE, and central nervous system (CNS) infection, such as meningitis and possible CIE. His mental status failed to improve despite increasing the lactulose dosage to maintain adequate therapeutic daily bowel movements for HE. Repeat head CT and chest x-ray revealed no acute changes. The patient’s renal function remained stable back to his baseline CKD ranges (1.8 mg/dL). HE was thought to be a less likely cause, given the fact that the patient had high stool output and low ammonia level (18 µg/dL). A random cefepime level was checked and found to be significantly elevated at 95 µg/mL (normal range: 5-10 µg/mL). At this point, CIE was suspected, and cefepime was discontinued in lieu of meropenem.

**Figure 1:**
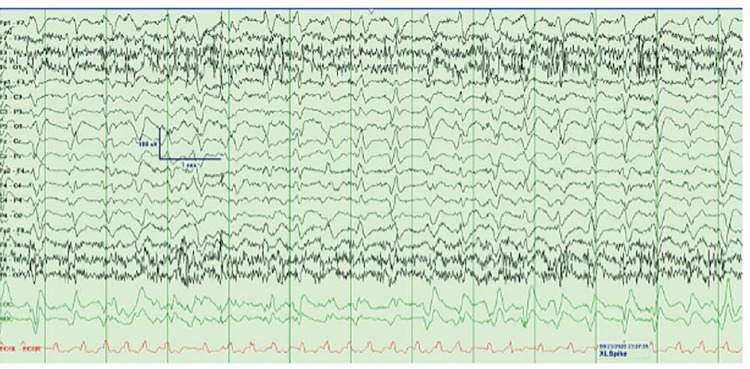
EEG finding consistent with tri-phasic wave Continuous generalized periodic discharges fluctuating into a rhythmic delta pattern and evolving in frequency to 2 Hertz. Morphologically, these discharges had a triphasic morphology with a moderate amplitude, sharp negative component followed by broader moderate amplitude, positive component followed by lower amplitude negative slow waveform consistent with a tri-phasic wave.

**Figure 2:**
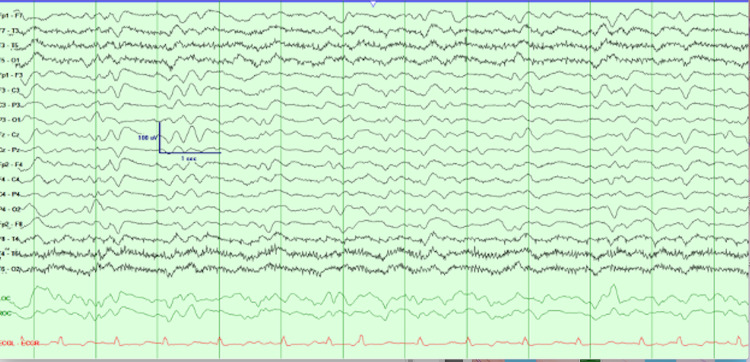
EEG finding consistent with dependent tri-phasic waveforms following anti-convulsive administration The rhythmicity seen in previous EEG abated after administration of levetiracetam. And there were some state-dependent triphasic waveforms.

In addition, an EEG was performed showed epilepsy with a triphasic wave most consistent with non-convulsive status epilepticus (NCSE) (Figure 1). Anti-seizure medications were administered; however, the patient was no longer able to protect his airway that warranted intubation. A repeat EEG demonstrated some improvement of NCSE following anticonvulsant administration (Figure 2). After cefepime was stopped, there was improvement noted in the patient’s mental status for several days; he was back to his baseline alert and oriented to time and place, and follows comments. The patient was extubated successfully and transferred to the medical ward for further management. The remarkable improvement of the patient’s mental status following cefepime discontinuation supported the diagnosis of CIE. Throughout his hospital course, the patient’s overall conditions began to deteriorate again, he was reintubated and went into multi-organ failure attributed to decompensated heart failure and ventilator-associated pneumonia with sepsis required prolonged intubation and pressor support which collectively resulted in the patient’s death.

## Discussion

Literature review

**Figure 3:**
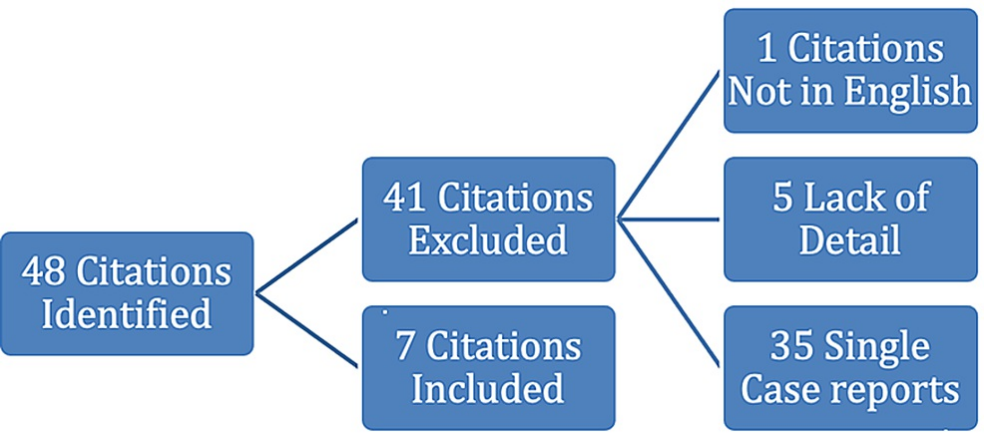
Characteristics of systemic review of the literature and articles included in our manuscript

To improve the clinical importance and understanding of CIE in the hospital setting, a comprehensive systematic literature review was performed using the search terms cefepime, neurotoxicity, and encephalopathy. The advanced search option extracted all CIE cases published in the English language. We identified a total of 48 articles that provided updated information regarding epidemiological findings, clinical information, diagnosis, and treatment of CIE. Single case reports, literature published in other languages, and studies that lacked detail of updated epidemiological findings were excluded (Figure 3). As outlined in Table 1, only seven studies met our search criteria and were selected to be published in this article. Based on this literature review, the rates of CIE have been found steadily increasing, potentially due to greater clinical awareness of cefepime’s side effects in patients with advanced renal dysfunction.

**Table 1:** Summary of seven case series and retrospective studies that provide detail and updated epidemiological findings, clinical information, diagnosis, and treatment of CIE CIE - cefepime-induced encephalopathy

Authors	Study type	Aim of the study	Epidemiological viewpoints	Associated comorbidities and risk factor	Clinical presentations	Response to Cefepime withdrawal	Treatment and alternative antibiotics use	Major conclusion
Schlidt et al. [8]	Case series	To report five cases of cefepime-induced encephalopathy within one year at a single institution.	Incidence of CIE is likely much higher than 3%, as reported in other studies	Acute and chronic renal failure, and ESRD dialysis dependent	Weakness, Confusion, Difficulty Ambulating	Full recovery of all 5 patients following discontinuation of cefepime	Cessation of cefepime, using alternative antibiosis; Ertapene, and meropenem,	The incidence of CIE is likely underestimated
Payne et al. [7]	A systemic review of 37 studies	To characterize the clinical course of cefepime neurotoxicity and response to interventions.	Up to 25% CIE occurs in patients who receive really adjusted dosing	Risk factors include renal dysfunction, excessive dosing, preexisting brain injury, and elevated serum cefepime concentrations.	Reduced consciousness (47%), myoclonus (42%), confusion (42%).	Symptom improvement occurred in 89% of patients, and 87% survived hospital discharge	Intervention, which included cefepime discontinuation, antiepileptic drugs, administration, and hemodialysis.	CIE can occur despite appropriate dosing, usually resolves with drug interruption, but may require antiepileptic drug administration or dialysis
Fugate et al. [9]	A retrospective study	To characterize cefepime neurotoxicity in the ICU	This was not a prospective study of all patients in the ICU receiving cefepime, and thus we cannot estimate the true incidence of cefepime neurotoxicity in that population	Renal failure in any form was present in 84% patients. CKD affected 40%and 77% had AKI	Impaired consciousness (n = 13), myoclonus (n = 11), disorientation (n = 6), and NCSE (n = 1).	72 patients (69.9%) died, Of those 19 patients (26%) received cefepime	Dose adjustment and cefepime discontinuation	Critically ill patients with chronic kidney disease are particularly susceptible to cefepime neurotoxicity
Appa et al. [10]	A systemic review	To review the literature and provide clinicians with an evidence-based framework with which to recognize cefepime neurotoxicity	Single-center estimate of incidence was 1 in 480 courses of cefepime	Most, patients had renal dysfunction (87%), and approximately one-third of subjects had end-stage renal disease	Diminished level of consciousness (80%), disorientation/agitation (47%), myoclonus (40%) NCSE (31%) and convulsive seizures seen only in (11%)	13% of subjects died during the same hospital stay in which they experienced neurotoxicity.	Cessation of cefepime was essential to recovery in all non-mortal cases, antiepileptic drugs were used in 33% and dialysis in 14%	Cefepime neurotoxicity should be considered in older patients with renal dysfunction and new onset encephalopathy. More work is needed to prospectively assess incidence and outcomes related to cefepime neurotoxicity
Jeon et al. [11]	A retrospective study	To report the development of CIE at a tertiary medical center in Korea and to describe the clinical features of CIE.	The incidence of CIE was 2.5% in this study	22.7% (14) of patients had CKD which 4/14 required dialysis	Confusion (36.4%), drowsiness (34.1%) stupor (20.5%), and comatose (9.1%)	The CIE patients showed full recovery after stopping cefepime.	Cefepime discontinuation, antiepileptic administration, and hemodialysis	It is essential to consider the possibility of CIE occurring in patients with renal failure or ESRD
Husari et al. [12]	A retrospective multicenter observational study	To investigate and evaluate CIE and to report the associated clinical, EEG expression with triphasic waves and the radiologic findings.	Previous reports suggest that 10% to 20% of patients develop CIE, however, a recent study from Taiwan reported only a 0.27% incidence rate	Eighteen patients (67%) had either acute kidney injury or chronic kidney disease or both	Myoclonus, (44%) and seizure before obtaining the EEG (4%)	All patients improved with discontinuation of cefepime.	Cefepime discontinuation, benzodiazepine administration	Cefepime toxicity should be considered in the differential diagnosis in encephalopathy patients with TWs
Ugai et al. [13]	A retrospective cohort study	To investigate the clinical features of patients with CIE.	Single-center cumulative incidence was reported to be approximately 4.1%	Acute renal failure, CKD, and ERSD requiring dialysis was significantly associated with the development of CIE	The most common clinical manifestations were decreased level of consciousness and myoclonus.	Symptoms resolved fully in all patients.	Cefepime discontinuation, 3 patients were switched to ciprofloxacin, 2 switched to piperacillin – tazobactam, and 1 switched to meropenem	This study indicated that the development of CIE is associated with severely impaired renal function in patients with HM

Additionally, to emphasize careful administration of cefepime in patients with advanced liver disease, a similar literature search was undertaken. There were a couple of cases found in the literature, which were summarized in Table 2.

**Table 2:** To date reported cases of CIE in patients with advanced liver diseases

Authors	Age/Gender	Underlying comorbidities	Clinical manifestation	Treatment	Response to therapy	Outcome
De Silva et al. [14]	71 years old, male	Alcohol-related liver cirrhosis	Confusion, altered mental status	Cefepime cessation	Resolved	To report a case of CIE in the setting advanced liver disease with normal renal function and unchanged baseline ammonia level and total bilirubin level
Kamboj et al. [15]	41 years old, female	Alcohol-related liver cirrhosis	Confusion, coma	Intubation, cefepime discontinuation	Marked improvement following cefepime discontinuation within 24 hours	To report a case of CIE in a patient with liver cirrhosis and intact renal functions. CIE was diagnosed after patient refractory HE and other possible causes excluded
Current case	67 years old, male	Decompensated liver cirrhosis, CKD stage IV, CHF	Confusion, coma	Intubation, Cefepime discontinuation, anti epileptic medications	Not improved, the patient passed away due to worsening AMS and subsequent respiratory failure	CIE should be highly suspected in critically ill patients particularly in the setting of advanced liver and renal diseases.

Cefepime, a fourth-generation cephalosporin antibiotic, is known for its broad antimicrobial activity including its efficacy towards Pseudomonas. Because cefepime is predominantly (85%) renally excreted, the possibility of increased retention in the bloodstream is greater in those with decreased renal function, leading to neurotoxicity [7]. The true incidence of CIE is unknown but could be as high as 15% in critically ill patients but is likely underreported. The first case of CIE was reported in 1999 in a patient with ESRD [16]. Since then, many reports have focused on understanding the pathophysiology, correlated risk factors, clinical manifestations, and the treatment options of CIE.

The pathophysiology of cefepime-induced neurotoxicity is still not fully understood; however, it is believed it could be related to the concentration-dependent competitive γ-aminobutyric acid (GABA) inhibition [16]. In pre-clinical studies, cephalosporins were shown to decrease GABA release from nerve terminals and increase excitatory amino acid release [17]. Based on these findings, cefepime neurotoxicity is thought to induce hyperexcitation of the neurons and depolarize the postsynaptic membrane, which as a result can lead to seizures, myoclonus, and coma [18].

**Figure 4:**
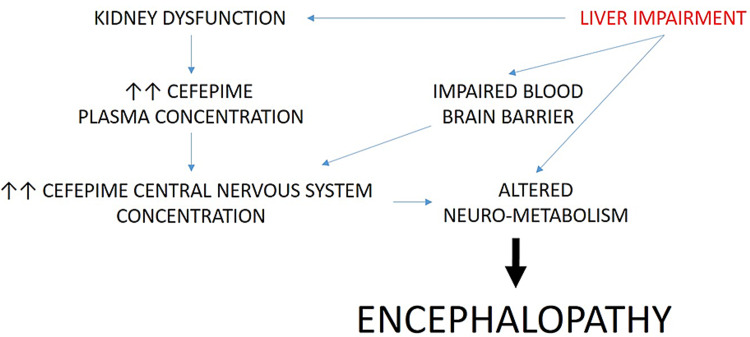
Pathophysiology of cefepime-induced encephalopathy in the setting of kidney and liver dysfunction

Renal failure is a well-known risk factor for cefepime toxicity given the fact that cefepime is predominantly excreted through kidneys. In a prospective cohort study, the incidence of CIE in patients with any medical illness was only 1%; while patients with renal impairment (GFR ≤ 15 mL/min) were found to have an increased incidence of 4.5% to 16.6% [19]. It is important to note, that CIE can occur in patients with a normal renal function as well despite appropriate cefepime dosing [20,21]. Besides renal dysfunction, other risk factors for CIE include excessive dosing, preexisting brain injury, old age, and hypoalbuminemia [7,22,23]. Furthermore, a disruption in the integrity of BBB from any causes such as sepsis, CNS infection, uremia, or a previous brain injury can increase the risk of CIE due to an increase in CNS penetration of cefepime of up to 45% (10% in normal conditions) [24,25]. A less recognized risk factor for CIE could be hepatic impairment. Liver dysfunction can lead to hypoalbuminemia, BBB disturbance, kidney dysfunction, altered neurotransmission, and neuro-inflammation, which as a result can increase the risk of CIE. There are two case studies reported in the literature that concluded that hepatic impairment could also increase the risk for CIE [14,15]. However, a retrospective comparing 243 patients without CIE to 10 patients who developed presumed CIE, did not identify hepatic impairment as a potential risk factor for CIE in a univariate analysis. In that study, hepatic impairment was defined as total bilirubin above 3 mg/mL or three times above upper limit elevation in transaminases. Only one patient in the CIE group had hepatic impairment [26]. This study is therefore underpowered to conclude that liver dysfunction is not a risk factor for CIE. The exact pathophysiological role of liver dysfunction in CIE is unclear since cefepime is metabolized by the liver minimally (<1%) [7]. It is believed that reduction in serum albumin levels, increased permeability of the BBB, hepato-renal syndrome, and neurotoxin accumulation increase the risk of CIE in those patients (Figure 4). Hence, it is essential to keep CIE as a differential diagnosis in any patients with the aforementioned risk factors.

CIE remains diagnosed of exclusion due to its unspecific manifestations. Distinguishing CIE from other causes of neurotoxicity is challenging and requires a high index of suspicion, especially in patients with underlying liver disease. Signs and symptoms of CIE are non-specific that can range from a reduced level of consciousness to seizures. The most common symptoms reported in patients with CIE are weakness, altered mentation, myoclonus, NCSE, and the symptoms can occur up to 10 days of cefepime initiation with a median of four to five days [7]. In a study by Fugate et al., CIE was suspected in 15% of ICU patients treated with cefepime for a minimum of three days [27]. As supporting evidence, EEG findings might be helpful in making a diagnosis [18]. As seen in a 2019 retrospective chart review which evaluated the EEG findings of 42 patients with CIE, one of the most frequent EEG findings was NCSE (64%), while generalized periodic discharge with or without triphasic morphology was the second most common EEG pattern (38%) followed by generalized rhythmic delta activity and generalized spike-and-waves [28]. However, these EEG findings are not specific and they cannot rule in CIE, because these EEG patterns can be seen in several other conditions such as HE [7]. Cefepime discontinuation and dose reduction are the mainstay treatment for CIE. The majority of patients have a favorable response to discontinuation of cefepime and resolution can usually occur at a median of two days. Additional interventions such as antiepileptic drug administration or dialysis might be required in severe cases [12].

In the present case, our patient was critically ill, had stage IV CKD along with cirrhosis. After cefepime was stopped, there was improvement noted in the patient’s mental status which lasted for several days, that strongly supported the diagnosis of CIE. Given the fact that our patient had significant other underlying comorbidities, his overall conditions began to deteriorate again, had worsening AMS and he went into multi-organ failure mainly attributed to decompensated heart failure and ventilator-associated pneumonia with sepsis requiring prolonged intubation and pressor support which collectively resulted in patient’s death.

The patient in our case was critically ill with significant underlying other comorbidities as described above. Therefore, multifactorial causes of the patient's AMS cannot fully be excluded. 

## Conclusions

CIE is a challenging diagnosis, involving the interplay of several organ systems. Systemic inflammation, renal impairment, and liver dysfunction can all lead to a vicious cycle of disruption of the BBB and altered neurological metabolism, which as a result increases the risk for CIE. Therefore, once other etiologies are ruled out, CIE should be highly suspected in patients with AMS and concomitant use of cefepime. Besides renal dysfunction, critical illness, systemic infection, and pre-existing neurological disease, hepatic impairment should be considered a risk factor for CIE. An EEG should be performed immediately for the diagnosis and cefepime should be discontinued. Consideration of alternative antibiotics should be used if no other etiology has been established.
